# Imaging vascular characteristics and glycolytic metabolism of glioblastoma in a chick embryo model using ^1^H MRI and [^18^F]FDG-PET

**DOI:** 10.1007/s11307-026-02084-x

**Published:** 2026-02-23

**Authors:** Elisabeth Non Gash, Jan Schulze, Sarah E. Barnett, Mahon L. Maguire, Michael Batie, Mohesh Moothanchery, Stephen Pickup, Ian Scott, Rasheed Zakaria, Judy M. Coulson, Sonia Rocha, Harish Poptani

**Affiliations:** 1 Department of Molecular & Clinical Cancer Medicine, Institute of Systems, Molecular & Integrative Biology, University of Liverpool, Liverpool, UK; 2 Egg Facility, Liverpool Shared Research Facility, University of Liverpool, Liverpool, UK; 3 Centre for Pre-Clinical Imaging, Liverpool Shared Resource Facilities, University of Liverpool, Nuffield Wing, Sherrington Building, Crown Street, Liverpool, L69 3BX UK; 4 Department of Biochemistry and Systems Biology, Institute of Systems, Molecular & Integrative Biology, University of Liverpool, Liverpool, UK; 5 Department of Radiology, University of Pennsylvania, Philadelphia, Pennsylvania USA; 6 Department of Pathology, The Walton Centre NHS Trust, Liverpool, UK; 7 Department of Neurosurgery, Walton Centre NHS Foundation Trust, Liverpool, UK

**Keywords:** Chick chorioallantoic membrane, Glioblastoma, MRI, PET, Metabolism, Vasculature

## Abstract

**Purpose:**

To assess hypoxia-associated host-tumour vascular adaptations and glycolytic metabolism in the chick chorioallantoic membrane (CAM) glioblastoma model.

**Procedures:**

U251 GBM cells were conditioned under normoxia (21% O₂) or hypoxia (1% O₂) for 72 h before implantation onto the CAM on embryonic day 7 (E7). Imaging was performed on E13 using MRI (control-CAM n = 8, normoxic-tumour n = 7, hypoxic-tumour n = 6) and brightfield microscopy (control-CAM n = 7, normoxic-tumour n = 8, hypoxic-tumour n = 7). Tumours were harvested on E14 for histology and gene expression analyses. In a separate cohort of 25 GBM-CAM tumours grown under normoxic conditioning, the correlation of glucose metabolism was assessed using [^18^F]FDG-PET on E12 followed by lactate MRS on E13 (n = 8).

**Results:**

Normoxia- and hypoxia-conditioned tumour-bearing CAMs exhibited vascular remodelling and significant upregulation of *VEGFA* and *ADM* compared to cultured cells. αSMA staining confirmed vessel infiltration in normoxia-conditioned tumours. CAIX staining revealed a hypoxic core in these tumours while hypoxia-conditioned tumours displayed heterogeneous staining. In both conditions, GLUT1 staining colocalised with CAIX staining, indicating hypoxia-associated glycolysis. *GLUT1, PDK1* and *LDHA* expression was elevated in CAM tumours relative to tumour cells *in vitro.* In the metabolic imaging cohort, most tumours exhibited [^18^F]FDG uptake and lactate signal. However, no statistically significant relationship was observed between the two methods.

**Conclusions:**

The CAM model provides a versatile platform for investigating GBM vascularisation and metabolism. Hypoxic conditioning amplifies transcriptional and vascular changes to the CAM. Although both [^18^F]FDG uptake and lactate were measurable, no significant correlation between the two was observed, potentially reflecting variability in tumour engraftment, vascular delivery of [^18^F]FDG, and microenvironmental influences on lactate accumulation.

## Introduction

Glioblastoma (GBM), also referred as WHO grade 4 glioma [1], is the most common and aggressive malignant brain tumour in adults. It is characterized by rapid proliferation, diffuse infiltration, and resistance to conventional therapies [2]. In comparison to low-grade glioma, the hypoxic tumour microenvironment plays a critical role in driving GBM malignant progression [3]. Hypoxia promotes vascularisation and induces metabolic rewiring, shifting tumour cells towards glycolytic metabolism to sustain growth and survival under oxygen-deprived conditions [4].

Hypoxia leads to the stabilisation of hypoxia-inducible factor α (HIF-α), which orchestrates a cellular transcriptional programme to promote tumour adaptation and progression [5]. A major consequence of HIF-α activation in GBM is the induction of pro-angiogenic signalling, which compensates for reduced oxygen supply by stimulating neovascularization in and around the tumour. HIF-α upregulates expression of proangiogenic genes such as vascular endothelial growth factors (*VEGFA*), angiopoietins (*ANGPT*), platelet-derived growth factors (*PDGF*), and adrenomedullin (*ADM*), which promote endothelial cell proliferation, migration, and survival [6]. HIF-α-mediated upregulation of matrix metalloproteinases (*MMPs*) facilitates extracellular matrix remodelling, enabling endothelial sprouting and expansion of the vascular network [7]. The resulting vasculature is abnormal; newly formed blood vessels are disorganized, tortuous, and highly permeable due to defective pericyte coverage and endothelial junction instability. This further exacerbates intratumoural hypoxia, sustaining HIF activation [8]. In addition to promoting angiogenesis, GBM cells can also alter the host vasculature through vessel co-option, allowing for tumour cell invasion alongside host blood vessels without requiring new capillary formation [9].

**Fig. 1:**
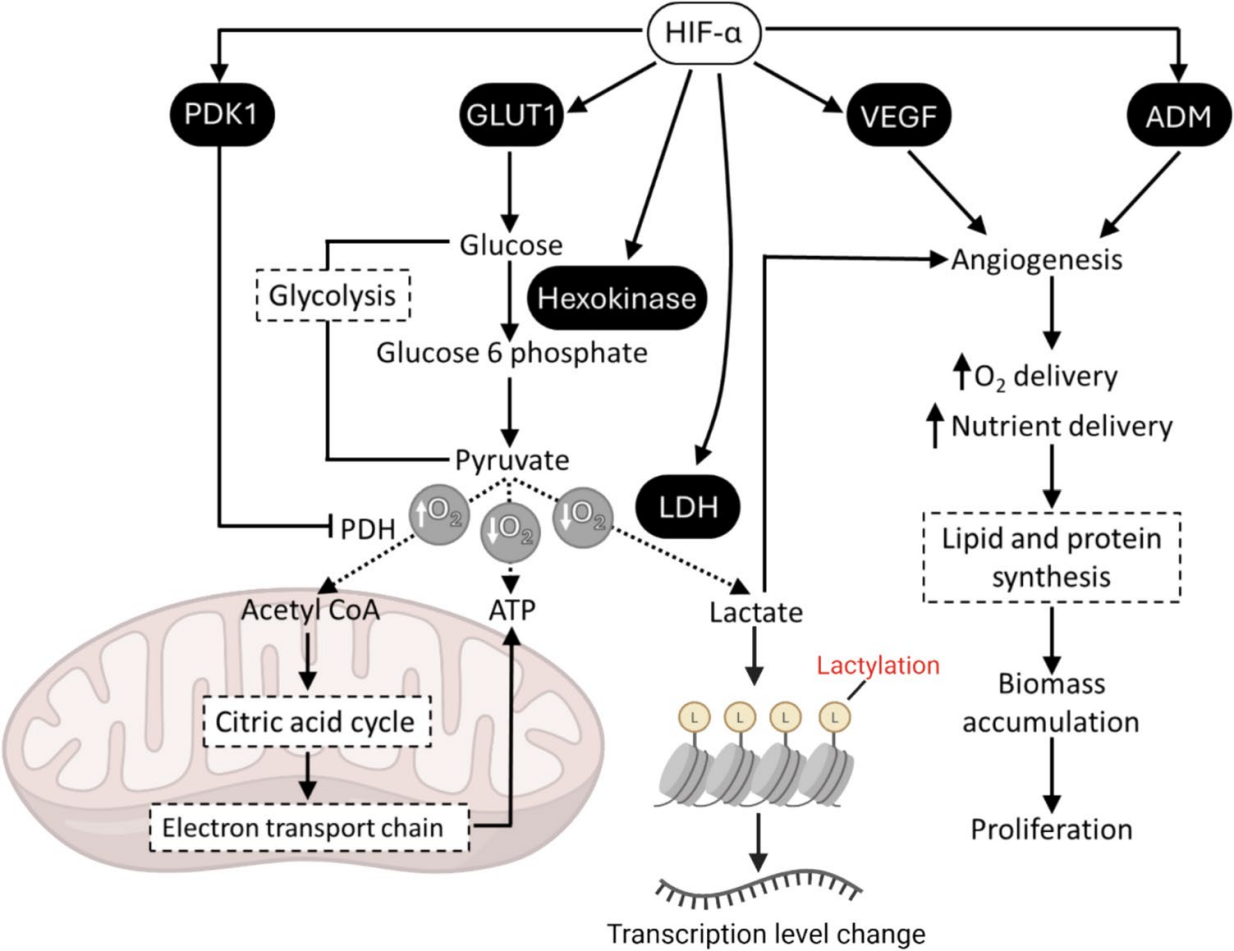
Pro-glycolytic and pro-angiogenic transcriptional roles of HIF-α. HIF-α upregulates pyruvate dehydrogenase kinase (PDK), which phosphorylates and inactivates pyruvate dehydrogenase (PDH), thereby reducing its active form and limiting acetyl-CoA production. Additionally, HIF-α enhances the expression of glucose transporters (GLUT) on the cell membrane, increasing glucose uptake to sustain glycolytic metabolism. This metabolic shift leads to increased conversion of pyruvate to lactate via lactate dehydrogenase (LDH), whose expression is also upregulated by HIF-α. Lactate efflux, mediated by monocarboxylate transporter 4 (MCT4), plays an essential role in maintenance of pH homeostasis during such metabolic adaptations. Elevated intracellular lactate serves as a substrate for protein lysine lactylation. Lactylation has been implicated in the regulation of gene expression under hypoxia, potentially influencing chromatin accessibility and HIF targeted gene transcription. In parallel, HIF-α promotes the transcription of VEGF and ADM, promoting angiogenesis for greater oxygen and nutrient delivery to support biomass synthesis and cellular proliferation. Figure adapted from [13] and used with permission

The hypoxia-driven angiogenic response in GBM is closely linked with metabolic adaptations that support tumour survival. HIFs shift cellular metabolism towards glycolysis, in which glucose is converted to lactate (Warburg effect), facilitating energy production in the absence of oxygen (Fig. 1) [10]. This meets the increased energy demand of rapidly dividing tumour cells and enables survival in hypoxic microenvironments through bypassing the oxygen-dependent tricarboxylic acid (TCA) cycle. Consequently, the switch to glycolytic metabolism is characterised by high glucose uptake, mediated by increased expression of glucose transporter 1 (*GLUT1* (gene name Solute Carrier Family 2 Member 1 (*SLC2A1*)) and elevated lactate production, via lactate dehydrogenase A (*LDHA*). In addition to its well-established role as a metabolic end-product, lactate has recently been implicated in the epigenetic regulation of gene expression via histone lactylation, a post-translational modification [11]. This emerging mechanism may serve as a metabolic-epigenetic link, particularly under hypoxic conditions where glycolytic flux is heightened [12].

Metabolic imaging facilitates non-invasive detection of glycolytic markers and, since changes in tumour metabolism can occur early during treatment, these markers may provide a powerful tool to detect early evidence of treatment response. PET/CT measurements of [^18^F]fluoro-D-glucose ([^18^F]FDG) uptake are widely used clinically for tumour staging, prognosis and treatment monitoring [14–16]. Upon injection, [^18^F]FDG enters the cells through glucose transporters, where it is phosphorylated and trapped, with higher signal intensity on the images reflecting elevated glucose uptake. Similarly, proton magnetic resonance spectroscopy (^1^H MRS) is a well-established non-invasive technique for monitoring tumour metabolism, based on the distinct signal profiles of individual metabolites. Increased signals from lactate, as well as choline and lipids are typically observed in GBM [17].

Rodent models of cancer have played a critical role in imaging studies of tumour biology and for development of novel drugs prior to clinical assessment. However, these models impose substantial financial and practical husbandry requirements and have an extended lag-time to establish xenografts (2–8 months). Additionally, variability in xenograft uptake across animal strains adds complexity, often limiting reproducibility and accessibility [18, 19]. The complexity of rodent GBM models makes it challenging to study tumour-host vascular interactions in a controlled manner, creating a need for alternative *in vivo* platforms that allow investigation of GBM pathophysiology and rapid evaluation of treatment responses alongside clinical therapies.

The chick chorioallantoic membrane (CAM) model has recently gained attention as a bridging model between *in vitro* and *in vivo* studies [20, 21]. The CAM is a highly vascularized, extraembryonic membrane surrounding the developing chick embryo, providing a naturally immunodeficient environment that readily supports tumour engraftment [22]. Importantly, its well-defined and accessible vascular network enables controlled investigation of tumour-host vascular interactions, while also supporting *in vivo* imaging for real-time analysis of vascular and metabolic dynamics [23]. Furthermore, CAM vessels are suitable for radiotracer injection, and several CAM studies have explored various radiotracers, cancer models, imaging techniques, and comparisons to mouse models [24–31].

Despite the known links between hypoxia, aberrant vasculature and glycolysis, they have rarely been studied together in the same model system. In this study, we investigated how hypoxia influences GBM growth in a CAM model through two critical facets of the tumour microenvironment: host-tumour vascular dynamics and metabolic reprogramming. While distinct, these processes jointly sustain tumour growth and therapeutic resistance. We utilised brightfield microscopy and MRI to assess hypoxia-induced host-tumour vascular remodelling. We also used clinically relevant imaging, [^18^F]FDG-PET and a lactate-selective MRS sequence—selective multiple quantum coherence (SelMQC)—to measure glycolytic metabolism in GBM xenografts in the CAM model.

## Materials and Methods

**Fig. 2:**
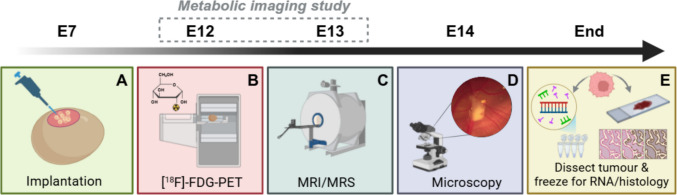
Experimental workflow of GBM-CAM imaging. **A**: On embryonic day 7 (E7), the tumour is implanted on the CAM. **B**: For the metabolic imaging study, eggs are imaged with [^18^F]FDG-PET on E12, and **C**: with MRI and MRS on E13. **D**: On E14 the eggs are imaged using microscopy and terminated. **E**: Tumours are dissected from the CAM and fixed/frozen for RNA or histological analysis. Figure created with BioRender.com

The experimental workflow used in the study is briefly summarized in Fig. 2.

### Cell lines and cell culture

The human GBM cell line U251 (RRID: CVCL_0021) was maintained in Glutamax (Gibco, Waltham, MA, USA) with 10% foetal bovine serum (Gibco, Waltham, MA, USA) and penicillin and streptomycin (100 mg/ml, Thermo Fisher Scientific, Cheshire, UK), in a 5% CO_2_ humidified incubator at 37 °C. The cells were split at 80% confluency every 3–4 days and regularly tested for mycoplasma (Lonza MycoAlert, Manchester, UK). For hypoxia studies, cells were pre-incubated in a humidified 1% O₂/5% CO₂/94% N₂ environment for 72 h in an InVivO_2_ 300 workstation (Baker Ruskinn, Bridgend, UK), maintained at 37 °C.

### CAM xenograft generation

Tumour xenografts were generated as previously described [32]. Briefly, fertilised chicken eggs (Medeggs Ltd, Fakenham, UK) were incubated at 37 °C and 45% humidity to initiate embryonic development (embryonic day 0; E0) in a BrinseaOvaEasy poultry incubator (Brinsea, Cheshire, UK). 2 × 10^6^ U251 cells were pipetted onto the CAM on E7 (Fig. 2A). On E14, tumours were dissected and experiments terminated in accordance with the UK Animals (Scientific Procedures) Act 1986 (amended 2012).

### [^18^F]FDG-PET/CT

PET and CT imaging was performed using a β-CUBE and X-CUBE (Molecubes, Ghent, Belgium) as previously described [31]. Briefly, on E12, [^18^F]FDG (5 ± 1 MBq in a final volume of 100–150 µL, Alliance Medical Radiopharmacy, UK) was injected distal to xenografted tumours into a large blood vessel of the CAM using a 33 G 12 mm hypodermic needle (Meso-relle; UKMedi, UK). The eggs (n = 25) were incubated for ~ 30 min after injection, ensuring complete uptake and distribution of [^18^F]FDG (as denoted in Fig. 2B).

### Magnetic resonance imaging and spectroscopy

MRI was performed on E13 using a horizontal bore 9.4 T Bruker Biospec 94/20 USR (Bruker, Ettlingen, Germany) system equipped with 440 mT/m imaging gradients (Fig. 2C). To minimise image artifacts arising from embryonic motion, eggs were pre-cooled on ice for 90 min prior to scanning, as previously described [32, 33], after which they were placed in a custom-built cradle. An 86 mm inner diameter quadrature volume coil was used for signal transmission/reception (Bruker, Ettlingen, Germany). High-resolution 3D images of CAM tumour and vasculature (control (CAM only) n = 8, tumour n = 7, hypoxia-preconditioned tumour n = 6) were acquired using a 3D TurboRARE (spin-echo, T_2_-weighted) pulse sequence with the following parameters: field of view 40 × 40 × 2 mm^3^, matrix size 256 × 256 × 12, voxel size = 156 × 156 × 167 µm^3^, TR/TE = 2000/45.81 ms, ESP = 9.162 ms, RARE factor = 14, slab thickness = 2 mm, 4 averages, flip angle = 90°, acquisition time = 33 m 36 s.

For the metabolic imaging study, Image-Selected *In vivo* Spectroscopy with Selective Multiple Quantum coherence (ISIS-SelMQC) [34] was performed on a separate cohort of eggs on E13 following [^18^F]FDG-PET imaging. MR images were acquired for voxel placement within the tumour, with a 3D TurboRARE (spin-echo, T_2_-weighted) pulse sequence with the following parameters: field of view 40 × 40 × 2 mm^3^, matrix size 267 × 267 × 14, voxel size = 156 × 156 × 167 µm^3^, TR/TE = 1000/69.52 ms, ESP = 9.162 ms, RARE factor = 14, slab thickness = 2 mm, 1 average, flip angle = 90°, acquisition time = 8 m 48 s. MRS sequence parameters included: TR = 2000 ms, 8012.82 Hz bandwidth, flip angle excitation/inversion = 90°/180°, 2048 complex points, averages = 4, acquisition time 8 m 32 s. Voxel dimensions were adjusted according to tumour size, ranging from 1.8—12.5 mm^3^.

### Microscopy

On E14, eggs were imaged with brightfield microscopy (Fig. 2D) using a Leica M165FC fluorescence stereomicroscope with 16.5:1 zoom optics, fitted with a Leica DFC425 C camera (Leica Biosystems, Wetzlar, Germany).

### Data analysis

The PET data were reconstructed, co-registered with the CT image and quantified using Invicro Vivoquant (Invicro LLCm, MA, USA, RRID:SCR_025778). A 3D ROI was manually drawn around each xenografted tumour to quantify the concentration of [^18^F]FDG. The SUVsum representing the total radiotracer uptake within the ROI, provides a measure of overall FDG accumulation in the tumour [24]. For MR images, Amira image analysis software (v9, Thermo Fisher Scientific, UK, RRID:SCR_007353) was used to manually segment and determine the volume of tumour xenografts and vessels within a region of interest of 10 × 10 × 2 mm^3^ to ensure a consistent and objective measurement between different eggs. Lactate and water ISIS-selMQC spectra were quantified using TopSpin (Bruker, RRID:SCR_014227) by integrating their respective peak areas, and relative lactate (RelLac) was calculated as the lactate-to-water integral ratio. For brightfield microscopy, the IKOSA CAM Assay (v3.1.0, Kolaido, Thal, Switzerland) was used to determine relative blood vessel area, thickness (mm), length (mm) and number of branching points for the control tumour and hypoxic tumour-bearing CAM. The output data were normalised to the analysed area (mm^2^), excluding the tumour region in tumour-bearing CAMs.

### Histology and immunostaining

Dissected CAM tumour samples were collected at E14 and placed in 1 mL 10% neutral buffered formalin (Sigma, St. Louis, MO, USA) for 16 h and then transferred to 70% ethanol. The samples underwent automated tissue processing and embedding into paraffin blocks and sectioned at 4 µm onto SuperFrost Plus slides (Thermo Fisher Scientific, Warrington, UK). H&E staining was carried out for assessment of tissue architecture. To assess tumour hypoxia, vascularisation and glucose transporter expression, immunohistochemical staining was performed on the automated Leica BOND RXM using the following primary antibodies: Carbonic Anhydrase IX (CAIX) (D47G3; Cell Signalling Technology; 1:200 pH9), Alpha Smooth Muscle Actin (αSMA) (AB5694; Abcam; 1:200 pH9), GLUT1 (D3J3A; Cell Signalling Technology; 1:200 pH9). Diaminobenzidine (DAB) was used to visualise antibody binding, and haematoxylin counterstain to distinguish human tumour and chick cell nuclei. Sections were mounted with DPX mountant (Sigma, St. Louis, MO, USA). Slides were imaged using a digital slide scanner (Leica Aperio GT450 digital slide scanner). Aperio ImageScope software (Leica Microsystems Ltd, UK) was used for viewing images.

### RNA extraction and gene expression analysis

Immediately after dissection on E14, tumours (n = 9) were rinsed in ice cold Dulbeccos phosphate buffered saline (DPBS) (Thermo Fisher Scientific #14,190,094) before transferring to RNAlater (Ambion, Life Technologies, Carlsbad, CA, USA). Total RNA was extracted from tumours, as well as from cell lines cultured *in vitro*, using a peqGOLD total RNA kit (VWR Life Sciences). RNA was converted to complementary DNA using iScript reverse transcriptase kit (BioRad, Watford, UK). qRT-PCR was performed using iQ sybr green supermix (Bio-Rad, UK) or power-track SYBRGreen master mix (Applied Biosystems, UK) on the QuantStudio™ 1 System (Thermo Fisher Scientific, UK) with 96-well plates. Β-Actin was used as a normalising gene, and results were analysed using the ∆∆Ct method [35]. Target primers used for gene expression analysis are listed in Supplementary Table S1. All primers were designed to be human-specific for evaluating the human GBM U251 xenograft without transcript amplification from chick CAM tissue. Analysis was performed using QuantStudio™ Design and Analysis Software Version 1.5.2 (Thermo Fisher Scientific, UK).

### Statistical analysis

Statistical analyses were performed in GraphPad Prism version 10.4 for Windows (GraphPad Software, San Diego, CA, USA, RRID:SCR_002798). Spearman’s Rank correlation coefficients were calculated to assess the correlation between SUVsum and relative lactate. For brightfield microscopy and MR image data, the Shapiro–Wilk test was used to determine whether data were normally distributed, and analysis performed using parametric or non-parametric tests as appropriate. For image data with three conditions or more, statistical significance was determined using one-way ANOVA with post-hoc Tukey multiple comparison. *p*-values less than 0.05 were considered significant.

## Results

### GBM xenografts remodel the CAM vasculature

**Fig. 3:**
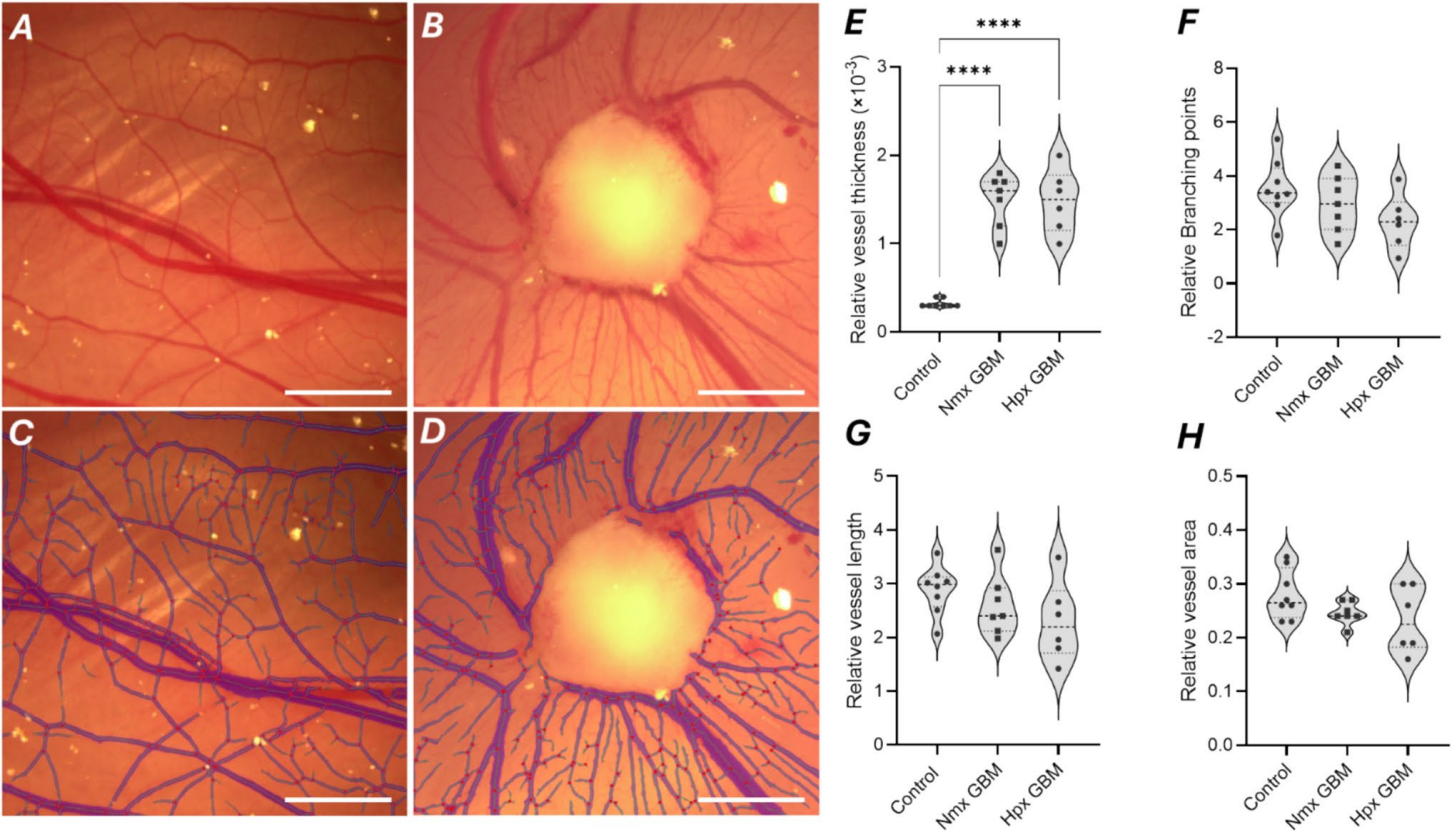
GBM tumours remodel the CAM vasculature. Representative brightfield microscopy images of control CAM (**A**) and tumour-bearing CAM (**B**) *in ovo*. The IKOSA CAM Assay mask (Kolaido CAM assay KML Vision) of the control (**C**) and tumour-bearing CAM (**D**). Scale bar: 2 mm. ROI: 10 × 10 mm.^2^. Violin plots of (**E**) relative vessel thickness, (**F**) branching points, (**G**) vessel length and (**H**) vessel area (relative to ROI analysed, excluding tumour region in tumour-bearing CAM images) for untreated non-tumour bearing (Control; n = 8), normoxia-preconditioned tumour-bearing (Nmx GBM; n = 7) and hypoxia-preconditioned tumour-bearing CAM (Hpx GBM; n = 6) output from the IKOSA CAM Assay. One-way ANOVA with Tukey’s post-hoc test., **** p < 0.0001

Brightfield microscopy images revealed that GBM tumours remodelled the CAM vasculature from a typical linear branching pattern to a radial ‘spokes of a wheel’ pattern around the tumour nodule (Fig. 3A-3D). Relative vessel thickness was significantly higher in both normoxia- and hypoxia-preconditioned tumour-bearing CAMs compared with CAM controls (Control CAM vs. Nmx GBM: p < 0.0001; Control CAM vs. Hpx GBM: p < 0.0001), with no difference between Nmx GBM and Hpx GBM (p = 0.99) (Fig. 3H). There were no significant differences between the three conditions for the other vessel parameters: relative vessel area (Control CAM vs. Nmx GBM: p = 0.32; Control CAM vs. Hpx GBM: p = 0.16; Nmx GBM vs. Hpx GBM: p = 0.87), number of branching points (Control CAM vs. Nmx GBM: p = 0.54; Control CAM vs. Hpx GBM: p = 0.09; Nmx GBM vs. Hpx GBM: p = 0.5), and mean vessel length (Control CAM vs. Nmx GBM: p = 0.6; Control CAM vs. Hpx GBM: p = 0.17; Nmx GBM vs. Hpx GBM: p = 0.63) (Fig. 3E-G).

**Fig. 4:**
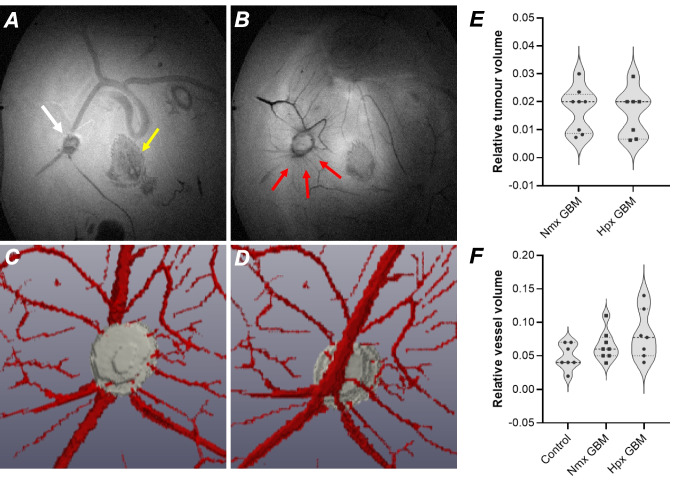
MRI enables 3D in-ovo visualisation of the tumour-CAM vasculature relationship. Representative MR image slices in the coronal plane, moving up towards the surface of the CAM (**A**, **B**). These images were chosen to focus on the tumour and its feeding vessels. The tumour (white arrow) and the associated vessels (red arrows) appear dark, the CAM appears as grey. The chick embryo organs are also observed as variable intensities, with the spine and flank of the chick observable near the centre of the image (yellow arrow). (**C**, **D**) Representative segmented masks, top and bottom view, used to determine tumour and vessel volume. (**E**) Violin plots showing relative tumour volume, calculated as tumour volume/total ROI volume, for normoxia (Nmx GBM; n = 8) and hypoxia-preconditioned (Hpx GBM; n = 7) CAM tumours, and (**F**) relative blood vessel volume for non-tumour bearing CAM (Control; n = 7), normoxia (Nmx GBM; n = 8) and hypoxia-preconditioned (Hpx GBM; n = 7) tumour-bearing CAMs.

Since brightfield microscopy only provides a two-dimensional (2D) cross sectional view of the CAM, we performed MRI to obtain 3D volumetric measurements of the tumour and CAM vessels using high-resolution images of the tumour and surrounding host vasculature (Fig. 4A). As with brightfield microscopy images, a radial pattern of CAM vasculature could be observed with MRI, with small vessels appearing to sprout from larger CAM vessels (Fig. 4B). Tumour volumes were similar between normoxia and hypoxia-preconditioned tumours (Fig. 4E). There were no statistically significant differences in the relative vessel volume between CAM and normoxia-conditioned tumour-bearing CAMs, or between normoxia and hypoxia-conditioned tumour-bearing CAMs (Control vs. Nmx GBM: p = 0.48; Control vs. Hpx GBM: p = 0.08; Nmx GBM vs. Hpx GBM: p = 0.49) (Fig. 4F).

### GBM-CAM xenografts exhibit hallmark metabolic switch to glycolysis

**Fig. 5:**
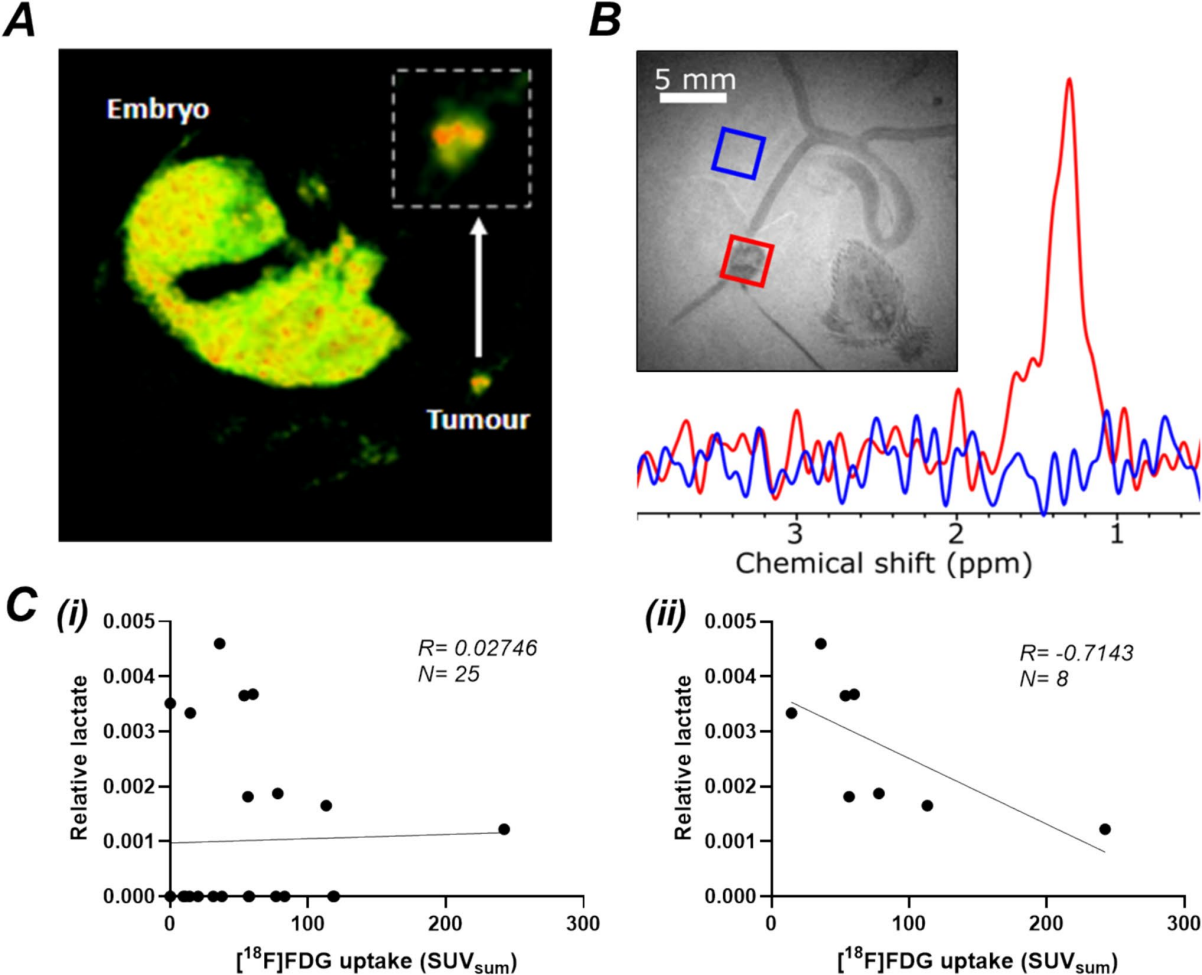
[^18^F]FDG uptake and lactate levels in GBM. (**A**) Representative image of [^18^F]FDG uptake into the chick embryo and tumour after injection of 5 ± 1 MBq [^18^F]FDG into a large vessel on E12. FDG signal is visible from the chick embryo and the GBM. The white arrow points to an inset, where an enlarged image of the same tumour is shown to reflect the heterogeneity in the FDG uptake within the tumour. (**B**) SelMQC sequence demonstrated a clearly visible lactate resonance from the tumour (red), but not from the CAM (blue). (**C**) Spearman’s Rank correlation between relative lactate and FDG uptake (SUVsum​) shown for (i) all tumours, including 0 values (R = 0.161, p = 0.441, n = 25), and (ii) both detectable PET and lactate signals (R = −0.71, p = 0.058, n = 8).

[^18^F]FDG-PET imaging was performed on E12 followed by MRS on E13 (n = 25). [^18^F]FDG uptake was observed in the tumour with notable uptake in the chick embryos (Fig. 5A), reflecting successful radiotracer injection and the high metabolic demand required for embryonic development. Lactate was detected in the CAM xenografts (Fig. 5B). MR spectra acquired from the tumour and adjacent CAM regions demonstrated differential metabolite signals, with tumour regions showing a prominent lactate peak at ~ 1.3 ppm (Fig. 5B, Figure S3A,C), whereas spectra from the CAM exhibited no lactate signal (Figure S3B,D). Some tumours had undetectable FDG uptake or lactate signal, and zero values were assigned to these cases for analysis. Examples of tumours that exhibited or lacked [^18^F]FDG uptake are shown in Figure S1. H&E and immunohistochemical staining for vasculature (αSMA), hypoxia (CAIX) and glucose transporter expression (GLUT1) was also performed to evaluate metabolic marker expression and assess tumour viability and engraftment quality. Histological analysis revealed that some tumours were detached from the CAM (Figure S2), indicating poor engraftment, which likely limited [^18^F]FDG delivery to these regions.

[^18^F]FDG uptake and relative lactate values are summarised in Supplementary Table S2. No correlation was observed between FDG uptake and lactate (Fig. 5Ci) when zero values were included. In order to avoid the impact of zero-clustered data, we performed the correlation excluding the zero values, and in this case again, there was no significant correlation between FDG and lactate, however the shape of the correlation trended negative (Fig. 5Cii).

### GBM-CAM xenografts form a hypoxic core

**Fig. 6:**
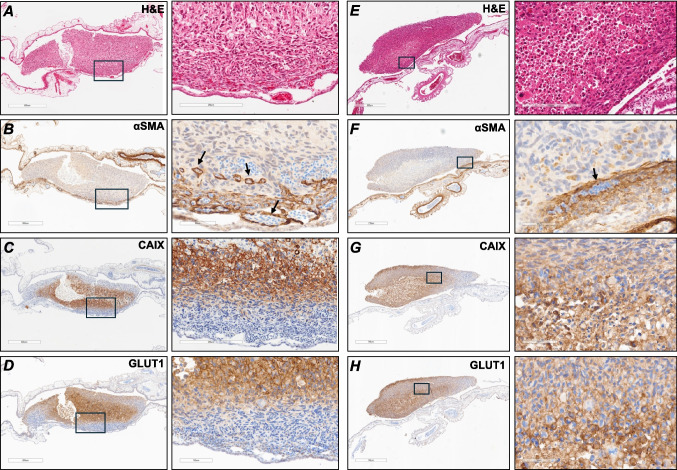
Histological characterisation of GBM-CAM tumour morphology, vascularisation, hypoxia and glucose transport. H&E staining of normoxia-preconditioned (Nmx) (**A**) and hypoxia-preconditioned (Hpx) (**E**) U251 tumours (scale bars: 600 μm, 200 μm), focussing on the tumour-CAM interface. αSMA staining revealed blood vessels in the CAM and at the periphery of both Nmx (**B**) and Hpx (**F**) tumours. Nucleated chick blood cells appear as blue dots inside the blood vessels and spaces between tumour cells (scale bars: 600 μm, 60 μm). CAIX staining showed hypoxic tumour cells localised to the centre of Nmx tumour (**C**), surrounding a necrotic core, whereas the Hpx tumour (**G**) exhibited heterogenous staining throughout the whole tumour (scale bars: 800 μm, 200 μm). GLUT1 staining was localised to the central region of the Nmx tumour (**D**), while the Hpx tumour (**H**) displayed a heterogeneous pattern that mirrored CAIX distribution (scale bar: 800 μm, 100 μm). Note the CAM has folded over the tumour in the case of the Nmx tumour

Vascularised tumour nodules formed on the CAM in most cases (Fig. 6). However, in some instances, vascularised tumours developed within the CAM, beneath the initial site of implantation (Figure S4). H&E staining revealed viable, differentiating tumour cells (Fig. 6A). IHC staining for the endothelial marker αSMA revealed large vessels in the CAM and at the tumour periphery in both normoxia- and hypoxia-preconditioned tumours (Figs. 6B and 6 F). Normoxia-preconditioned xenografts showed strong CAIX staining concentrated in the centre of the tumour, surrounding a region in the core lacking nuclei, indicative of necrosis (Fig. 6C). In contrast, hypoxia-preconditioned tumours exhibited a more heterogeneous CAIX distribution throughout the tumour mass (Fig. 6G), suggesting a sustained hypoxic phenotype driven by ‘hypoxic memory’, as previously described [36]. GLUT1 staining strongly colocalised with CAIX staining in both normoxia- and hypoxia-preconditioned tumours (Fig. 6D and H).

### GBM-CAM tumour exhibits upregulation of proangiogenic and proglycolytic genes

**Fig. 7:**
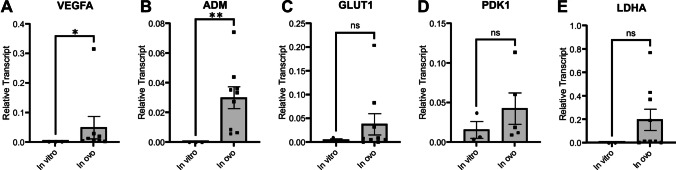
GBM-CAM xenografts demonstrate higher expression of HIF-α target genes involved in angiogenesis and glycolytic metabolism relative to cells in culture. qPCR analysis of U251 cells cultured *in vitro* and normoxia/control-conditioned CAM-tumour xenografts *in ovo*. **A**) *VEGFA* and **B**) *ADM* promote angiogenesis to restore oxygen and nutrient supply. **C**) *GLUT1* is involved in glucose transport. **D**) *PDK1* phosphorylates pyruvate dehydrogenase for ubiquitination, preventing conversion of pyruvate to acetyl-coA. **E**) *LDHA* catalyzes conversion of pyruvate to lactate. For the *in vitro* condition, a single biological replicate yielded no detectable Ct value for target gene *LDHA* and was therefore excluded from analysis. For the above genes, expression is elevated *in ovo* compared to *in vitro*. Mean expression is shown relative to the mean of β-actin (2.^∆Cq^). Bars represent the mean ± standard deviation (SD) of biological replicates, with individual data points shown. Unpaired t-test, * p < 0.05, ** p < 0.01. Supporting data for housekeeping genes is shown in Supplementary Figure S5

mRNA expression levels of proglycolytic and proangiogenic targets of HIF-α demonstrated elevated transcription in tumours compared with U251 cells grown in culture in normoxia for proangiogenic genes *VEGFA* and *ADM* (p = 0.02 and p = 0.01, respectively), and for proglycolytic genes *GLUT1*, *PDK1* and *LDHA*, although not statistically significant (p = 0.60, p = 0.37, p = 0.27 respectively) (Fig. 7). Housekeeping gene expression was stable across conditions, as Ct values for actin remained consistent between *in vitro* cell studies and CAM xenografts under both normoxic and hypoxic conditions (Figure S5).

## Discussion

In this study, we demonstrate that the U251 chick CAM model recapitulates several clinically relevant hallmark features of GBM. Hypoxic tumour cells within the xenograft core may be activating HIF-α signalling to promote angiogenesis and metabolic reprogramming. These phenotypes can be assessed using a range of complementary techniques, highlighting the versatility of the model for investigating both cellular and microenvironmental processes. Collectively, these attributes establish the CAM model as a physiologically relevant platform for studying tumour metabolism and vascular remodelling in GBM.

U251 xenografts remodelled the CAM vasculature (Figs. 3 and 4), in concordance with recent reports describing radial remodelling of blood vessels around mesothelioma cell line-derived CAM xenografts [32]. Relative vessel thickness was significantly higher in tumour-bearing CAMs compared to control CAMs. However, no significant difference was noted between control CAMs or tumour bearing CAMs vessel length, area or volume. In addition, no significant difference was observed between normoxia- and hypoxia-preconditioned tumours with regards to vessel thickness, vascular volume or tumour volume. This may suggest that 72 h hypoxic preconditioning does not exert an effect on the tumour-host angiogenic response beyond that of tumour grafting alone, although the 72 h hypoxic preconditioning did suggest ‘cellular hypoxic memory’ in GBM-CAM xenografts, as evidenced histologically (Fig. 6), whereby normoxic tumours showed a gradient of CAIX staining with a necrotic core, while the hypoxic tumours demonstrated a heterogenous CAIX staining throughout the tumour. Herrmann et al. (2015) [36] reported that the 72 h hypoxic preconditioning significantly increased the metastatic potential of neuroblastoma-CAM tumours. In addition, Al-Mutawa et al. (2018) [37] reported significant changes in the tumour cell metabolome, including high levels of ketones (3-hydroxybutyrate), lactate, and phosphocholine in neuroblastoma CAM tumours preconditioned in 72 h hypoxia in comparison to normoxic CAM tumours. These studies along with our findings suggest that the 72 h hypoxic conditioning retains the “hypoxic memory” even though such differences may not be observed macroscopically using the imaging methods used in our study. Future studies could benefit from exploring the effects of different durations of hypoxic preconditioning to ascertain the optimal timing for lasting epigenetic effects. [^18^F]FDG uptake and lactate signal demonstrated that GBM-CAM xenografts exhibited a glycolytic phenotype (Fig. 5). No significant correlation between these two measures was observed. However, the sample size following the exclusion of zero values (n = 8) may be too low to draw robust conclusions and should be regarded as exploratory data. Nevertheless, these findings challenge the assumption that higher [^18^F]FDG uptake would correspond with increased lactate production and provide grounds for future investigation with a larger sample size. Previous studies have investigated the relationship between [^18^F]FDG uptake and lactate levels, yielding varied results. In one study, Herholz et al. reported a significant positive correlation between lactate concentration and [^18^F]FDG uptake in patients with gliomas [38]. However, their study included a heterogeneous mix of glioma subtypes and grades and, in many instances, regions of elevated [^18^F]FDG uptake did not spatially coincide with areas of increased lactate, suggesting a lack of direct correspondence between [^18^F]FDG uptake and lactate production. Moreover, as the patients were undergoing variable treatments during imaging including corticosteroids or radiotherapy, the treatment itself could have confounded the observed correlation between FDG uptake and lactate concentration. On the other hand, Guo et al. reported no significant correlation between [^18^F]FDG uptake and relative lactate in patients with lung adenocarcinoma [39]. Here again, ongoing treatment at the time of imaging may have confounded the assessment of tumour-intrinsic metabolic activity. More recently, Van Heijster et al., using hyperpolarised [1-^13^C]pyruvate MRS in murine xenografted human prostate cancer cell lines, reported a significant negative correlation between [^18^F]FDG uptake and lactate production (measured as the pyruvate-to-lactate conversion rate) [40]. Collectively, these varied findings align with our results, indicating that increased [^18^F]FDG uptake may not necessarily be a predictor of elevated lactate production, but that the two modalities reflect distinct, partially overlapping facets of tumour glycolytic metabolism.

Several technical and biological factors could explain the lack of correlation between FDG uptake and lactate in our GBM-CAM xenografts. Firstly, [^18^F]FDG-PET and lactate MRS have very different sensitivities and detection limits. In the case of lactate, non-detectable signals may not necessarily reflect an absence of production, but rather the limited sensitivity of MRS combined with rapid vascular clearance or metabolic reutilisation of lactate. The ISIS pulse sequence is also particularly sensitive to motion, further contributing to apparent zero values. Secondly, [^18^F]FDG uptake is strongly dependent on tumour vascularisation, which determines both tracer delivery and clearance. Heterogenous vascular recruitment across xenografts may therefore underlie some of the variability in [^18^F]FDG signal, with poorly vascularised tumours showing minimal or no [^18^F]FDG uptake despite active glycolysis. Indeed, histological assessment of tumours in which no FDG uptake was observed, but in which lactate was detected, demonstrated poor overall engraftment, both in terms of attachment to the CAM and of vascular supply (Figure S2). Thirdly, [^18^F]FDG uptake itself is limited by the expression and activity of GLUT, as well as by hexokinase activity, meaning that high [^18^F]FDG accumulation may not necessarily equate to high glycolytic metabolism.

Furthermore, lactate is not merely a terminal byproduct influenced by glycolytic flux; its concentration is also influenced by downstream metabolic and microenvironmental processes. Lactate can be rapidly exported and cleared from the tumour via the vasculature or be metabolised through conversion back to pyruvate in order to fuel the TCA cycle [41, 42]. In addition, lactate may be diverted into alternative pathways such as histone lactylation [11, 43]. Thus, while both [^18^F]FDG uptake and lactate signal are indicative of glycolytic activity, they capture different aspects of tumour metabolism—glucose transport and phosphorylation versus steady-state lactate metabolism and clearance. The absence of a correlation between FDG uptake and lactate signal in our model may be reflective of such complexities.

Notably, in our dataset, the inclusion of samples with non-detectable lactate signal as well as non-detectable [^18^F]FDG uptake resulted in no correlation between [^18^F]FDG uptake and lactate. In contrast, exclusion of these apparent zero values produced a negative trend. The correlation plots highlight that the apparent relationship is strongly influenced by zero-inflated data from both modalities: for FDG-PET, zeros can arise from variable vascular delivery or transporter activity, while for lactate they may reflect MRS sensitivity, clearance, or reutilisation. Thus, the correlation outcome appears highly sensitive to methodological and detection thresholds across both readouts, rather than purely reflecting biological differences. Fundamentally, the exclusion of zero values reflects biological phenomena, while inclusion of zeros may incorporate technical artifacts related to variable vascular access.

Immunoreactivity of hypoxia-inducible molecular marker CAIX was localised to the centre of the tumour, demonstrating that GBM-CAM tumours develop a hypoxic core driven by an oxygen gradient similar to that observed in spheroid, rodent, and patient-derived GBM models (Fig. 6) [44–46]. The colocalization of GLUT1 and CAIX staining suggests a hypoxia-driven shift towards glycolytic metabolism. Furthermore, the absence of CAIX with correspondingly low levels of GLUT1 staining in tumour cells on the periphery of the tumour, or close to intratumoural vessels, suggests an oxygen supply adequate to support aerobic respiration thereby preventing activation of the hypoxic response. This reasoning is corroborated by hypoxia-preconditioned tumours displaying reduced expression of GLUT1 and CAIX staining at the site in which the tumour appears integrated with the vascularised CAM (Fig. 6, G&H). GBM-CAM tumours also exhibited elevated mRNA levels of canonical pro-angiogenic HIF-α targets (*VEGFA* and *ADM)* compared with cultured cells, supporting the hypothesised hypoxia-induced transcriptional reprogramming within GBM-CAM tumours (Fig. 7). However, the mRNA expression of key glycolytic genes (*GLUT1, LDHA, and PDK1*) was not significantly higher in CAM xenografts than in cultured cells. Given the major role HIF plays in the transcriptional upregulation of glycolytic proteins [46], together with our positive immunohistochemical staining for GLUT1 and CAIX, this may indicate additional HIF-mediated post-transcriptional control of glycolytic enzyme expression [47]. Future work could investigate non-canonical HIF pathways of regulation in GBM-CAM tumours to clearly delineate the role of hypoxia in this model. 

While the chick CAM offers a partial solution to the cost, husbandry, timescale and ethical issues posed by rodent models, it imposes some unique constraints. In the UK for example, the CAM tumour model can persist until E14 before legal restrictions from the Animal (Scientific Procedure) Act apply. This imposes a restricted timeframe, presenting challenges for studying tumour progression, vascular remodelling, and treatment responses, which can be followed over extended periods in mammalian models. Additionally, seasonal changes and temperature extremes, particularly in the winter and summer, can negatively affect embryo viability leading to inconsistent survival rates. Such limitations can partially be mitigated by starting experiments with larger numbers of eggs.

Beyond egg viability, intra- and inter-batch variability presents another challenge. Even within the same shipment, embryos may develop at slightly different rates, affecting tumour engraftment, vascularization, and response to experimental conditions. To account for this, experimental design should be kept simple to avoid additional variables and be powered accordingly to account for survival and engraftment rates. Standardized protocols for egg handling, incubation, and tumour implantation improve reproducibility, but some degree of biological variability remains inevitable. By accounting for these challenges through careful experimental design, the CAM model remains a powerful tool for preclinical research, particularly for rapid screening of tumour-host dynamics and therapeutic interventions.

In order to reduce egg mortality and minimise motion artefacts associated with warming during prolonged scan times, the MR-derived vascular volume measurements were obtained in a cohort distinct from the eggs imaged by MRS and PET. Future work should ideally implement faster, integrated vascular imaging protocols to acquire all modalities in a single cohort, enabling direct pairing of vascular volume with metabolic readouts. Furthermore, methodological refinement to reduce motion could strengthen CAM MR imaging studies. MRI was performed following 90 min of cooling, as previously described [33]. However, recent work demonstrated that anaesthetising eggs with isoflurane in a sealed plastic bag can rapidly and effectively suppress motion artefacts [47]. Isoflurane anaesthetisation is widely used in rodent models, and, while associated with vasodilation [48, 49] and slowed metabolic rate [50], it may offer a preferable alternative to cooling, which could induce CAM vasoconstriction, hypothermic shock, stress signalling, and altered intratumoural metabolism. However, as cooling was applied consistently across all eggs, comparisons between conditions remain valid. While the impact of pre-cooling on embryonic stress responses or tumour biology has not been formally assessed, such effects are plausible. Future studies directly comparing cooled versus anaesthetised embryos could quantify the degree to which thermal stress influences metabolic and vascular metrics, as well as downstream mRNA and IHC readouts. 

In summary, this study demonstrates the utility of the GBM-CAM model for investigating hypoxia-driven metabolic reprogramming and tumour-induced vascular remodelling using molecular imaging techniques. The integration of [^18^F]FDG-PET, lactate MRS, brightfield vascular imaging, histology, and measurement of gene expression provides a comprehensive assessment of GBM cell xenografts in a physiologically relevant, cost-effective, and imaging-compatible model. These findings highlight the potential of the CAM for preclinical evaluation of metabolic imaging biomarkers and therapeutic strategies targeting the hypoxic tumour microenvironment.

## Data Availability

Upon acceptance, raw data will be made available through a data repository.
